# Effects of Acute Consumption of Noni and Chokeberry Juices vs. Energy Drinks on Blood Pressure, Heart Rate, and Blood Glucose in Young Adults

**DOI:** 10.1155/2019/6076751

**Published:** 2019-08-18

**Authors:** Dariusz Nowak, Michał Gośliński, Anna Wesołowska, Karolina Berenda, Cezary Popławski

**Affiliations:** Department of Nutrition and Dietetics, Faculty of Health Sciences, Ludwik Rydygier Collegium Medicum in Bydgoszcz, Nicolaus Copernicus University in Toruń, Bydgoszcz, Poland

## Abstract

The purpose of this study has been to determine the effect of acute consumption of noni and chokeberry juices vs. energy drinks on blood pressure, heart rate, and blood glucose. The subjects divided into 4 groups, which consumed three portions of noni or chokeberry juices (30 mL or 200 mL, respectively) and energy drink (ED) or water (200 mL) at one-hour intervals. All participants had their blood pressure (BP), both systolic and diastolic BP (SBP and DBP), as well as heart rate (HR) and blood glucose (BG), measured. Consumption of noni juice caused a significant decrease in SBP and DBP of 5.0% and 7.5%, respectively, while, the consumption of chokeberry juice slightly decreased only DBP by 3.6%. On the contrary, consumption of three portions of EDs caused a significant increase in DBP by 14.7%. The BG of participants consuming noni juice decreased by 7.3%, while the consumption of EDs increased BG by as much as 15.8%. Acute consumption of noni juice contributed to a significantly decreased SBP, DBP, and HR as well as a mild reduction of BG. Consumption of chokeberry juice caused only a slight reduction of DBP. Contrary to juices, EDs consumption resulted in an increase of blood pressure (especially DBP) and blood glucose. The results of the study showed that noni juice may be effective in lowering blood pressure and blood sugar levels, but there is a need to continue research on the long-term effect of this juice.

## 1. Introduction

The fruit of noni (*Morinda citrifolia* L.) originates from Southeast Asia (Polynesia and India), but it is also grown in South America and the Caribbean Islands [1]. Traditionally, juice extraction is preceded by fermentation, and juices are best made from 45-day-old fruit, which has the most beneficial content of bioactive compounds [1, 2]. Noni fruit juice (NJ) is roughly comparable to apple juice in its nutritional analysis although it is somewhat lower in calories. The reason is that noni fruit contains over threefold less total carbohydrates than apples do. Moreover, NJ is low in protein, calories, and fat and has no cholesterol. Noni is a fairly good source of vitamin C (53.2 mg/100 mL) and other antioxidants, and it is relatively high in potassium (ca. 150 mg/100 mL) [2, 3]. The potassium content of noni juice is similar to other common juices, e.g., orange juice, tomato juice [4], and chokeberry juice [5]. Potassium promotes a healthy heart rhythm, muscular contraction, nerve function, energy production, and fluid balance. Moreover, potassium helps to lower blood pressure by minimizing the effects of salt [2]. On the contrary, twelve compounds have been identified in noni juice, of which nine have been quantified, and these include phenolic compounds such as coumarins (scopoletin and esculetin), flavonoids (rutin, quercetin, quercetin derivative, isoquercitrin, and kaempferol), phenolic acid (vanillic acid), vanillin, and iridoids (asperulosidic acid and deacetylasperulosidic acid). Iridoids have been identified as the major compounds in noni juice, next to flavonoids such as rutin and scopoletin [6]. Noni juice has been found to contain some quantities of resveratrol and phenolic acids (mainly ferulic and caffeic acids) [7]. The presence of numerous bioactive compounds has justified the classification of *Morinda citrifolia* as one of the dietary components which have a positive impact on the cardiovascular system, especially preventing hypertension, atherosclerosis, and dyslipidemia [8, 9]. Its positive influence on human health caused that noni juice has been classified in the EU as “novel foods,” whereas in the USA, it is described as a “nutritional supplement.” Nevertheless, there are no scientific studies published that include noni consumption guidelines. Typically, it is recommended to consume 30–60 mL·NJ, but ill people are suggested to drink as much as 180 mL of noni juice [2].

Chokeberry is a plant which belongs to the family Roseaceae. Three species of chokeberry are distinguished: *Aronia arbutifolia*, *Aronia prunifolia*, and *Aronia melanocarpa*. Among berry plants, chokeberry is distinguished by its high polyphenol content, with a prevalent share being anthocyanins and proanthocyanidins [10]. Considering anthocyanins, chokeberry juice (ChJ) is the richest in cyanidin-3-galactoside and cyanidin-3-arabinoside. It also contains phenolic acids, mainly chlorogenic and neochlorogenic acids [11–13]. Compared to noni, the content of vitamin C in chokeberry is small (from 0.013 to 0.27 mg/g dry matter), but the potassium content is considerable among all bionutrients (up to 2.18 mg/g dry matter) [14]. Chokeberry has a higher antioxidant capacity than other berries, such as blackberries, raspberries, cranberries, or blueberries [15]. Similar observations had been previously reported by Slatnar et al. [16] and Kulling and Rawel [5].

Intake of fruits and berries may lower blood pressure (BP), most probably owing to the high content of polyphenols [17]. Conversely, it has been demonstrated that consumption of energy drinks (EDs) rich in caffeine and sugar may have an adverse effect on human health, mainly by raising arterial blood pressure and blood glucose level; in the long term, it increases the risk of cardiovascular diseases and type 2 diabetes [18–23]. Cardiovascular diseases (CVD), according to the most recent statistics, are responsible for 45% of all deaths (49% in women and 40% in men). Coronary heart disease and stroke are the major causes of death due to CVD [24].

The purpose of this study has been to determine the effect of acute consumption of noni and chokeberry juices vs. energy drinks on arterial blood pressure, heart rate, and blood glucose.

## 2. Materials and Methods

### 2.1. Subjects

The study was carried out on a group of volunteers, students of a private college (Bydgoszcz, Poland). Healthy volunteers (aged over 18 years) were recruited for the study. Exclusion criteria were as follows: cardiovascular disease, any medication affecting the cardiovascular system, diabetes, other chronic diseases, pregnancy, lactation, and regular alcohol intake. All subjects signed an informed consent after reading the purpose and schedule of the research. Originally, 96 volunteers were recruited. The participants were asked to abstain from drinking alcohol or taking any supplements (for at least 24 h prior to the experiment) and to exclude from their diet (for at least 12 h before the test) sources of caffeine such as coffee, tea, and cola type of drinks. In addition, the research participants were requested not to do any excessive physical activity prior to the test. Before the tests, all subjects were informed about the research objective and schedule, after which they signed a written consent form to participate in the study. Finally, 88 subjects took part in the study. Eight people did not participate (either did not arrive, failed to follow the guidelines, or were afraid of blood tests). Subjects were divided into 4 groups: 1st—consuming 30 mL noni juice (noni group); 2nd—consuming 200 mL chokeberry juice (chokeberry group); 3rd—consuming 200 mL energy drink (ED group); 4th—drinking 200 mL water (control group). The assignment of participants to each group was random, aided by a random number generation formula (an MS Excel application). Before the onset of the tests, the participants received verbal instructions.

### 2.2. Samples

Organic noni fruit and chokeberry juices, both a rich source of polyphenols, were used for the study. The juices were cold-pressed from whole fruits. Chokeberry originated from Poland, while noni came from French Polynesia. Juices underwent mild flow pasteurization (the temperature not exceeded 80°C) in order to preserve nutrients. The ED group was given to consume one of the most popular energy drink, which contained 32 mg of caffeine per 100 mL. The energy drink was purchased in a local grocery shop, but prior to the test, it was coloured with a food dye and got rid of carbon dioxide. The control group was served still water which had also been coloured with a food dye. Until the tests, all the drinks (juices, EDs, and water) were stored at room temperature. Moreover, juices and energy drinks were previously analyzed to determine their pH and antioxidant capacity (TP and DPPH). The pH of the tested samples of juices was measured with a glass electrode at room temperature. Antioxidant capacity and total polyphenol content assays were described in previously studies [7]. The information presented of energy, protein, carbohydrates, total lipids, fiber, caffeine, taurine, inositol, etc., is derived from the labels of the noni juice, chokeberry juice, and energy drink samples. All data are presented in Table 1.

### 2.3. Study Protocol

The study was carried out between May and June 2018. On the day of the tests, all participants had fasted measured blood glucose, blood pressure (systolic and diastolic), and heart rate. Next, each subject consumed three portions of the drinks (described in the Samples section) at one-hour intervals. The noni group drank 30 mL of NJ (in total, 90 mL); the chokeberry group consumed 200 mL of ChJ (in total, 600 mL); the ED group had 200 mL of energy drink (in total, 600 mL); and the control group consumed 200 mL of water (in total 600 mL). Each participant had their blood pressure (SBP and DBP) and heart rate (HR) measured four times, i.e., before having the first juice or ED and 1, 2, and 3 h after the consumption of each portion. BP and HR measurements were done in accordance with the guidelines of the European Society of Hypertension and European Society of Cardiology [24], and the recommendations by Kallioinen et al. [25] using comfort sphygmomanometer (previously validated) after the participants had been seated and had rested for at least 5 min [26]. All tests were performed twice at 1-2 min intervals at room temperature, on the arm of the nondominant hand. Wherever large differences were observed, the measurement was repeated once more. Between the intervals of consumption of juice and measurements, the participants did not have any physical activity. The blood glucose level was measured with a glucometer Optium XIDO (Abbott Diabetes Care Ltd., Witney, UK) before having the first drink (juice or ED) and 1 h after consuming the third portion. The same protocol was followed for the control group. The tests were preceded by an interview about the daily diet, including questions about consumption of caffeinated drinks and juices. The study was performed in accordance with the Helsinki Declaration, and the protocol was approved by the University Bioethical Committee (KB 294, 295/2018).

### 2.4. Statistical Analyses

All the results were statistically analyzed by calculating the mean and standard deviation. The interpretation of the results was performed in MS Excel 2010 Analysis ToolPak software (Microsoft, Redmond, WA, USA), with one-way analysis of variance (ANOVA) and Tukey's test as the post hoc test; *p* values lower than 0.05 were considered as significant.

## 3. Results

### 3.1. Participants

The tests were performed on young adults (average age 25 years; 49 female and 39 male participants), most having a normal BMI (79.5% of the participants). 15 subjects (17%) were overweight, and 3 people were underweight. Baseline characteristics of the study participants are shown in Table 2.

Prior to the tests, all participants were interviewed about their dietary habits, including daily intake of caffeinated drinks, juices, and nectars. The results showed that most of the subjects consumed fruit juices (53 people consumed fruit juices daily). Forty-nine subjects drank coffee every day, of which 23 people preferred brewed coffee and 19 chose instant coffee. Twenty-eight subjects admitted to regular drinking energy drinks and cola-type beverages.

### 3.2. Blood Pressure Measurements

**Figure 1:**
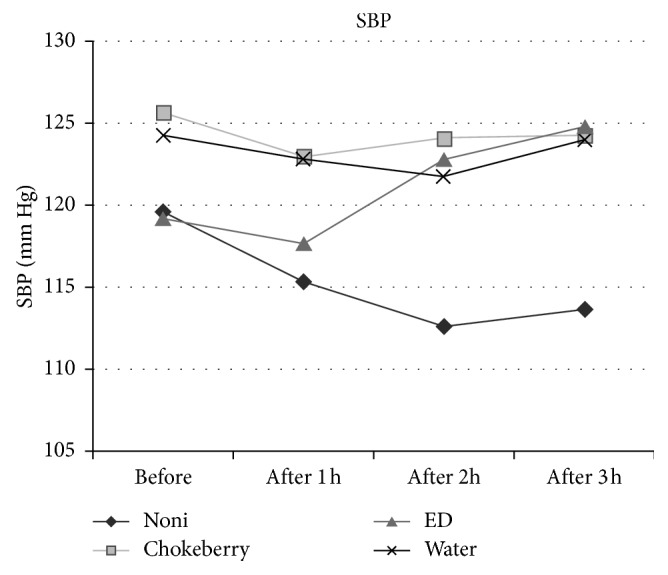
Changes in systolic blood pressure (SBP) of participants.

**Figure 2:**
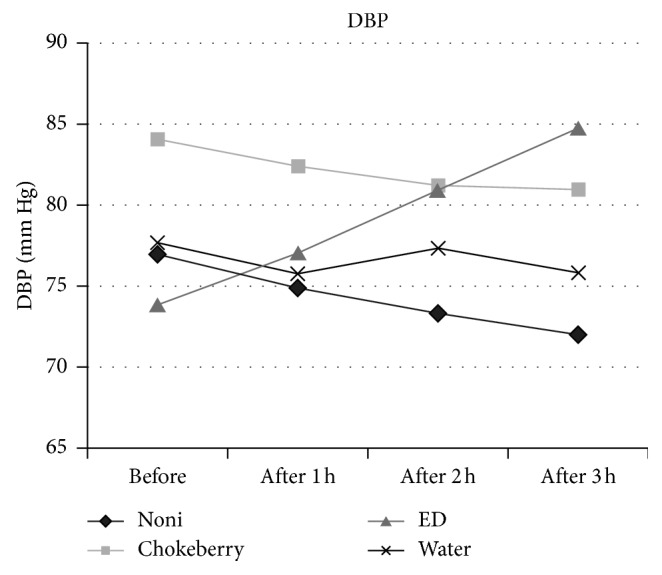
Changes in diastolic blood pressure (DBP) of participants.

**Figure 3:**
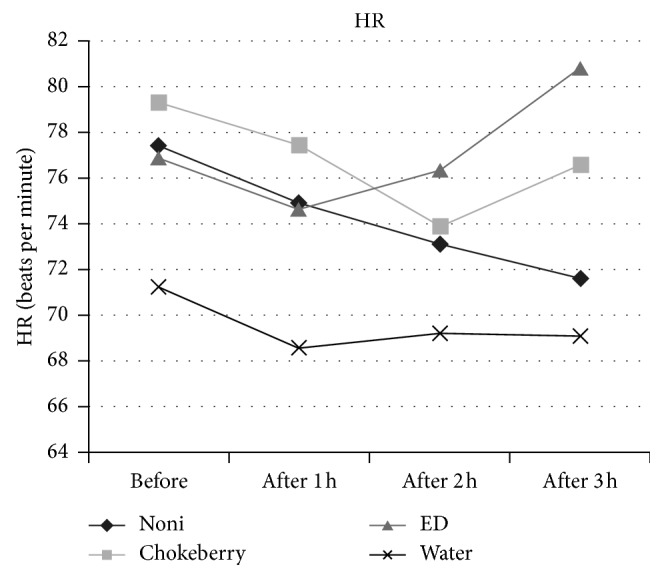
Changes in the heart rate (HR) of participants.

Results of the blood pressure measurements showed that the noni group had the highest decrease in both systolic and diastolic blood pressure, as well as heart rate. After drinking 30 mL of noni juice, the average SBP decreased by 3.8%, i.e., from 119.9 to 115.3 mm·Hg (Figure 1). After the next portion of NJ, the SBP fell down by 5.9% compared with the initial value, and after the third portion of NJ, the average SBP decreased statistically significantly (*p*=0.024) by 5.0%, i.e., down to 113.6 mm·Hg (Table 3). In the same group, the average DBP decreased considerably (*p*=0.006), from 77.0 to 72.0 mm·Hg, i.e., by 6.5%, after drinking three portions of NJ (Figure 2). After drinking 1 and 2 portions of noni juice, DBP decreased by 2.7 and 4.8%, respectively. The heart rate in subjects consuming NJ decreased by 3.2%, 5.6%, and 7.5% after each subsequent portion of the juice. The decrease in HR after the third portion of NJ was statistically significant (*p*=0.030) relative to the initial value (Figure 3). In contrast, the control group (consuming water) showed no statistically significant changes in SBP, DBP, and HR.

The chokeberry group (similar to the noni group) was observed to have decreased SBP and DBP as well as HR. The SBP decreased by 2.1% (i.e., from 125.6 to 123.0 mm·Hg) after consumption of the first portion of chokeberry juice (Figure 1). The subsequent portions of juice caused only a slight decrease by about 1–1.2% relative to the initial SBP, and these changes were not statistically significant (*p*=0.766). Slightly greater decrease owing to the consumption of chokeberry juice was observed in DBP, which was lowered by 1.8, 3.3, and 3.6%, respectively, after the first, second, and third portion of the juice. However, the decrease from 84.0 to 81.0 mm·Hg after drinking a total of 600 mL of chokeberry juice was not statistically significant (*p*=0.304) (Table 3, Figure 2). The values of HR in this group did not undergo a significant (*p*=0.507) decrease, as they fell down from 79.3 to 76.6 beats per minute.

Unlike the noni group and chokeberry group, the group consuming ED experienced an increase in the SBP, DBP, and HR after each portion of energy drink. Initially, having drunk the first portion of energy drink (200 mL), the participants had an unchanged SBP, while their DBP increased by 4.2% (Figures 1 and 2). After the second portion of ED, the average SBP increased slightly, by 3.0%, while the DBP rose considerably (by 9.4%). After the three portions of energy drink, the SBP increased statistically insignificant (*p*=0.206) from 119.2 to 124.8 mm·Hg (4.8%), while the DBP increased significantly (*p* < 0.001) from 73.9 to 84.8 mm·Hg (14.7%) (Table 3). The heart rate in this group did not undergo a significant (*p*=0.243) increase, rising from 76.9 to 80.8 beats per minute, even after consuming three portions of EDs.

### 3.3. Blood Glucose Measurements

**Figure 4:**
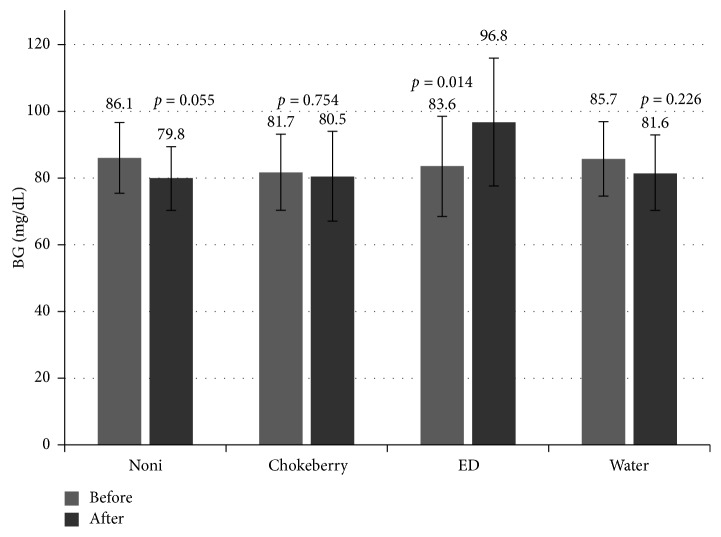
Values of blood glucose (mean) before and after consumption.

Further analyzes focused on the blood glucose levels. Consumption of three portions of NJ (each at 30 mL) caused decrease in BG by 7.3%, from 86.1 to 79.8 mg/dL (Table 3, Figure 4). Having consumed three portions of chokeberry juice (each at 200 mL), the BG of participants decreased slightly by 1.5%, from 81.7 to 80.5 mg/dL. Contrary to noni and chokeberry juices, consumption of 3 portions (each at 200 mL) of ED caused a significant (*p*=0.014) increase in BG, by 15.8%, i.e., from 83.6 to 96.8 mg/dL. In the control group (consuming water), the level of BG did not undergo any significant change (*p*=0.226).

## 4. Discussion

In our study, acute consumption of NJ (90 mL in total) caused a significant (*p*=0.024 and *p*=0.006) decrease in systolic and diastolic blood pressure, by 6.0 and 5.0 mm·Hg, respectively. Consumption of three portions (600 mL in total) of chokeberry juice resulted in a small decrease in SBP and DBP by 1.3 and 4.0 mm·Hg, respectively, which was not statistically significant (*p*=0.766 and *p*=0.304). In another pilot, crossover study [27], acute effects on blood pressure associated with consumption of a 300 mL anthocyanin-rich fruit juice were assessed. Young adults (*n* = 6) and older adults (*n* = 7) received in random order, either a single 300 mL dose or 3 × 100 ml doses of high-flavonoid cherry juice provided at 0, 1, and 2 h. Two hours after consumption of 1 × 300 mL of the juice, a decrease of SBP and DBP by 5.5 mm·Hg was observed, while the HR decreased by 4.8 beats per minute. The group consuming 3 × 100 mL of the juice was reported to have a decrease in SBP by 3.5 mm·Hg, DBP by 3.3 mm·Hg, and HR by 3.3 beats per minute. Blood pressure returned to the initial values 6 hours after drinking the juice. More favourable effects were observed in the first group, who drank a larger amount of juice on a single dose [27]. In yet another study, consumption of 59 mL of noni juice, twice per day for four weeks, decreased the mean SBP and DBP by 12 and by 7 mm·Hg, respectively [28]. There are also reports on research into effects of long-term consumption of polyphenol-rich juices, resulting in the reduction of arterial blood pressure. Skoczyńska et al. [29], who examined fifty-eight healthy men with diagnosed mild hypercholesterolemia without pharmacological intervention, concluded that consumption of 250 mL/day of chokeberry juice for six weeks caused a decrease in arterial SBP from 138.6 ± 19.1 to 130.4 ± 10.9 mm·Hg (*p* > 0.05), while DBP decreased from 89.0 ± 10.9 to 82.9 ± 6.2 mm·Hg (*p* < 0.05) [29]. In our study, blood pressure, especially SBP, decreased by 4.0 mm·Hg in response to acute consumption of chokeberry juice. Other researchers stated that consumption of grapefruit juice (for 6 months) reduced the stiffness of arteries without reducing blood pressure in postmenopausal women with normal arterial blood pressure [30]. Tjelle et al. [17] conducted an experiment which involved 2 groups of people aged 50–70 years: 1st group composed of patients with slightly elevated blood pressure (SBP 130–139 mm·Hg and DBP 85–89 mm·Hg) and 2nd group consisted of patients with hypertension (SBP 140–179 mm·Hg and DBP 90–109 mm·Hg). The research participants consumed polyphenol-rich multifruit juice for 12 weeks, 500 mL/daily, and had their blood pressure measured at the beginning of the experiment and after 6 and 12 weeks. The results showed that after 6 and 12 weeks, SBP in the 1st group decreased by 6.9 mm·Hg (after 6 weeks) and by 3.4 mm·Hg (after 12 weeks), whereas in the 2nd group, SBP decreased by 7.3 mm·Hg (after 6 weeks) and by 6.8 mm·Hg (after 12 weeks). These researchers concluded that a content of 11.9 mg polyphenols/100 g of juice was sufficient to reduce arterial blood pressure [17]. In our study, noni juice (353.4 mg of polyphenols per 100 g) caused a greater decrease in SBP, DBP, and HR than chokeberry juice with a higher content of polyphenols (1065.5 mg/100 g). This shows that the profile of bioactive compounds is more important than the total content of polyphenols. It is supposed that the beneficial effect of NJ on arterial blood pressure and blood glucose can be induced by specific flavonoids and iridoids. Dussossoy et al. [6] stated that iridoids, apart from flavonoids (especially rutin and scopoletin), are main compounds present in noni juice. As noted by Tjelle et al. [17], intake of fruits and berries may lower blood pressure, most probably due to the high content of polyphenols. Kent et al. [27] noted that anthocyanins reach the circulatory system in about 2 hours after consumption and are excreted in about 4 hours after a meal. This explains the cumulative effect of juices 3 hours after drinking their first portion. As well as being rich in polyphenols, noni juice contains much potassium, and therefore, 150 mL of this juice provides about 150 mg of this macronutrient, which contributes to the lowering of arterial blood pressure and prevents fatigue, muscle weakness, and spasm, and insomnia [2]. However, other researchers suggested that flavonoids of NJ play the major role in reducing blood pressure. Noni contains many rutin, quercetin, luteolin, and scopoletin (a coumarin derivative), which can lower blood pressure by increasing the activity of glutathione peroxidase and NO in the endothelial cells, causing vasorelaxation of blood vessels [31]. Moreover, these flavonoids showed inhibition effect on the activity of ACE (angiotensin-converting enzyme) [31–33].

In our research, consumption of three portions of noni or chokeberry juice decreased the HR by 5.8 and 2.7 beats per minute, respectively. In another study participated by young adults, 2 hours after drinking 300 mL (in a single dose, or in 3 portions at 100 mL) of juice rich in flavonoids, the heart rate decreased by 4.8 or by 3.3 beats per minute [27].

Contrary to the juices, increased SBP and DBP and HR were observed after consumption of three portions of EDs, by 5.6 and 10.9 mm·Hg, respectively, and by 3.9 beats per minute. In another study, higher SBP and DBP were also noticed 2 hours after drinking 355 mL of ED (114 mg of caffeine). The values increased by 5.2 and 6.1 mm·Hg, respectively [18]. There was no effect in the control group consuming water, neither in our study nor in the tests reported by Grasser et al. [18]. Other researchers have also demonstrated an increase in SBP and DBP, by about 6–10 mm·Hg and 3–6 mm·Hg, respectively, 1-2 hours after drinking EDs [19]. The observed rise in blood pressure and heart rate is a consequence of the presence of caffeine, sugar, and probably other substances in the composition of EDs. Regular consumption of these beverages can be hazardous, especially to adolescents, and in the long term can create a greater risk of suffering diabetes, obesity, and various cardiovascular disorders. We have demonstrated that consumption of three portions of EDs caused an increase in the average BG by 15% after 3 hours. In a previous study, the average rise in the blood glucose level due to the consumption of 3 portions of EDs was even higher, reaching 20.7% [23]. Also, other researchers showed that healthy young adults who consumed sugar-sweetened drinks with caffeine had a significant increase in BG and insulin levels after 20–30 min [22]. The consumption of EDs is very high among young adults. Therefore, noni or chokeberry juices can be an effective alternative for energy drinks causing decrease of blood pressure and blood glucose levels. Considering noni juice, three portions (each at 30 mL) caused a decrease of the final blood glucose by 7.3%, whereas consumption of three portions of chokeberry juice or water led to a decrease in BG by 1.5% and 4.8%, respectively. In another clinical trial, including 20 people, consumption of noni fruit juice in a dose of 2 mL per kg of body weight for 8 weeks resulted in a significant decrease in BG [34].

Studies on animals have also shown the efficiency of using NJ in lowering BG. Nayak et al. [35] have shown that a NJ dose of 2 mL/kg body weight twice a day decreased the fasting BG in streptozotocin-induced diabetic rats to 150 mg/dl compared with 300 mg/dl in controls. A marked decrease in BG was also observed in diabetic mice fed with fermented noni fruit juice [36]. In another experiment on mice, which were given NJ, the researchers noticed a decrease in BG and improved blood lipids. These authors conclude that noni juice can be helpful in prevention and treatment of diabetes [37].

In our study, the subjects drinking chokeberry juice were noticed to have an only slightly (*p*=0.754) lower level of BG, whereas Skoczyńska et al. [29] reported a considerable (*p* < 0.05) BG decrease, from 99.3 ± 14.5 to 91.8 ± 14.3 mg/dl. Other extracts containing flavonoids, especially anthocyanins, can decrease the BG. A study conducted on 10 overweight (BMI ≥ 25) people, aged 18–65 years, who consumed a drink made from acai berry in an amount of 100 g twice a day for 1 month, were found to have a BG lowered from 98.0 mg/dl to 92.8 mg/dl [38]. Animal model studies also provide similar observations. In a study on rats presenting diabetes mellitus induced by streptozotocin (100 mg/kg), daily intraperitoneal injections with the methanol extract of graviola leaves for two weeks caused a considerable decrease in BG from 21.64 to 4.22 mmol/l [39]. Likewise, administration of extract of goji berry (*Lycium barbarum* L.) to rabbits with diabetes (induced by alloxan) and hyperlipidemia resulted in a significant decrease in BG after ten days of experiment. The treatment also lowered total cholesterol and triglycerides in blood plasma but raised the level of HDL-C [40]. Similar effects were noticed in people who had consumed chokeberry juice [29].

The results of our study have proven that even acute consumption of noni juice, in small doses (3 × 30 mL), caused a considerable decrease in SBP, DBP, and HR, while inducing only a slight decrease in BG in young adults. Doses of 30 ml of noni juice are often recommended and have been used in previous studies [2, 9, 41]. Others used higher doses, i.e., 59 mL twice per day [42], or even more, i.e., 100 mL [43]. It is worth noting that daily consumption of as much as 750 mL of noni juice is safe for people [44]. Chokeberry juice was less effective although it was used in a larger amount than noni juice, but similar to other previous studies [29].

## 5. Conclusions

We have demonstrated that acute consumption of noni juice contributes to a significant decrease in both systolic and diastolic blood pressures as well as heart rate in young healthy adults. Moreover, NJ caused a slight decrease in the blood glucose. Consumption of chokeberry juice resulted in a decrease of DBP, but the changes were not statistically significant. The changes in SBP and HR were also insignificant. No statistically significant changes in BP and HR were observed in the control group consuming water. Contrary to the juices, the energy drinks contributed to a significant increase in DBP and BG but did not have a significant influence on SBP. Thus, consumption of EDs may induce an adverse impact on human health, whereas juices rich in polyphenols may play a beneficial role, especially by decreasing arterial blood pressure and blood glucose. These findings require further studies to explain the influence of long-term consumption of noni and chokeberry juices on the above parameters.

## Figures and Tables

**Table 1:** Characteristics of noni, chokeberry juices, and energy drink.

	Noni juice	Chokeberry juice	Energy drink
Energy (kcal/100 mL)	15	73	46
Protein (g/100 mL)	0.5	0	0
Carbohydrates (g/100 mL)	2.53	18.1	11
In it sugar (g/100 mL)	2.5	9.9	11
Total lipid (g/100 mL)	0.82	0.1	0
Saturated fatty acids (g/100 mL)	0	0.1	0
Fiber (g/100 mL)	0.1	0.1	0
Sodium (g/100 mL)	0	0	0.07
Caffeine (mg/100 mL)	—	—	32
Taurine (mg/100 mL)	—	—	400
Inositol (mg/100 mL)	—	—	20

pH	3.56^a^	3.54^a^	3.30^a^
Antioxidant capacity (DPPH) (mg·Tx/100 mL)	90.5^b^	448.6^a^	9.7^c^
Total polyphenols (TP) (mg·GAE/100 mL)	353.4^b^	1065.5^a^	31.4^c^

Tx: trolox equivalents; GAE: gallic acid equivalents. Statistical analysis was performed by one-way ANOVA using Tukey's post hoc test; different letters in the same row indicate statistical significance (at least at *p* ≤ 0.05).

**Table 2:** Baseline characteristics of the study participants (*n* = 88).

Parameter	Noni group	Chokeberry group	ED group	Control group	Average
Sex					
Female	15	10	13	11	**49**
Male	7	12	9	11	**39**
Age, years	25.9 ± 8.8^a^	25.2 ± 5.6^a^	25.2 ± 7.1^a^	23.7 ± 3.5^a^	**25.0** ± **6.5**
Height (m)	1.71 ± 0.10^a^	1.75 ± 0.10^a^	1.71 ± 0.08^a^	1.74 ± 0.09^a^	**1.73** ± **0.09**
Weight (kg)	67.7 ± 11.3^a^	70.2 ± 12.7^a^	68.6 ± 12.2^a^	69.7 ± 13.2^a^	**69.1** ± **12.2**
BMI	23.2 ± 3.5^a^	22.8 ± 2.7^a^	23.3 ± 3.8^a^	22.8 ± 2.7^a^	**23.0** ± **3.2**
>24.9	2	5	4	4	15
18.5–24.9	19	17	16	18	70
<18.5	1	0	2	0	3

Values are the mean ± standard deviation; different letters in the same row indicate statistical significance (at least *p* < 0.05).

**Table 3:** Blood pressure (SBP and DBP), heart rate (HR), and blood glucose (BG) of participants.

Parameter	Noni group (*n* = 22)			Chokeberry group (*n* = 22)			ED group (*n* = 22)			Control group (*n* = 22)		
	Before noni	After 3 noni	*p*	Before chokeberry	After chokeberry	*p*	Before ED	After 3 ED	*p*	Before water	After 3 water	*p*
SBP (mm·Hg)	119.6 ± 8.3	113.6 ± 8.5	0.024	125.6 ± 14.0	124.3 ± 16.1	0.766	119.2 ± 14.8	124.8 ± 14.1	0.206	124.3 ± 13.5	124.0 ± 11.4	0.933
DBP (mm·Hg)	77.0 ± 6.6	72.0 ± 4.8	0.006	84.0 ± 9.8	81.0 ± 9.9	0.304	73.9 ± 8.4	84.8 ± 9.9	<0.001	77.7 ± 9.2	75.8 ± 8.0	0.477
HR (beats per minute)	77.4 ± 9.0	71.6 ± 8.1	0.030	79.3 ± 13.3	76.6 ± 13.7	0.507	76.9 ± 10.7	80.8 ± 11.4	0.243	71.2 ± 9.9	69.1 ± 9.0	0.457
BG (mgdL^−1^)	86.1 ± 10.6	79.8 ± 9.5	0.055	81.7 ± 11.4	80.5 ± 13.3	0.754	83.6 ± 15.0	96.8 ± 19.1	0.014	85.7 ± 11.1	81.6 ± 11.2	0.226

Data are mean ± SD; statistical significance at *p* < 0.05. SBP: systolic blood pressure; DBP: diastolic blood pressure; HR: heart rate; BG: blood glucose.

## Data Availability

The data used to support the findings of this study are available from the corresponding author upon request.
